# Insights Into Dendritic Cells in Cancer Immunotherapy: From Bench to Clinical Applications

**DOI:** 10.3389/fcell.2021.686544

**Published:** 2021-06-28

**Authors:** Ahmed Salah, Hao Wang, Yanqin Li, Meng Ji, Wen-Bin Ou, Nianmin Qi, Yuehong Wu

**Affiliations:** ^1^Department of Biochemistry and Molecular Biology, College of Life Science and Medicine, Zhejiang Sci-Tech University, Hangzhou, China; ^2^Hangzhou Biaomo Biosciences Co., Ltd., Hangzhou, China; ^3^Asia Stem Cell Therapies Co., Limited, Shanghai, China

**Keywords:** dendritic cells, cancer vaccines, cancer immunotherapy, induced pluripotent stem cells, iPSC-DCs

## Abstract

Dendritic cells (DCs) are efficient antigen-presenting cells (APCs) and potent activators of naïve T cells. Therefore, they act as a connective ring between innate and adaptive immunity. DC subsets are heterogeneous in their ontogeny and functions. They have proven to potentially take up and process tumor-associated antigens (TAAs). In this regard, researchers have developed strategies such as genetically engineered or TAA-pulsed DC vaccines; these manipulated DCs have shown significant outcomes in clinical and preclinical models. Here, we review DC classification and address how DCs are skewed into an immunosuppressive phenotype in cancer patients. Additionally, we present the advancements in DCs as a platform for cancer immunotherapy, emphasizing the technologies used for *in vivo* targeting of endogenous DCs, *ex vivo* generated vaccines from peripheral blood monocytes, and induced pluripotent stem cell-derived DCs (iPSC-DCs) to boost antitumoral immunity.

## Introduction

Cancer evades immune surveillance as one of its hallmarks and prevents the immune system from tumor eradication (Hanahan and Weinberg, 2011; Mittal et al., 2014). Thus, immunotherapy, relying on cell therapy, cancer inhibitory signal antagonists, nanoparticle-based vaccines, oncolytic viruses, and immunogenic cell death-inducing agents, is considered a cornerstone in cancer treatment (Helmy et al., 2013; Yang, 2015; Kranz et al., 2016; Van der Jeught et al., 2018; Riley et al., 2019; Vanmeerbeek et al., 2020; Malvehy et al., 2021). In general, cell-based cancer immunotherapy can be divided into two subclasses, active and passive immunotherapies. Active immunotherapy utilizes antigen-presenting cells (APCs) such as dendritic cells (DCs) to boost patients’ immune system to fight against cancer (Van Lint et al., 2014; Jansen et al., 2020). However, passive immunotherapy mostly involves immunization with T cells to induce immune-mediated tumor rejection, including adoptive transfer of tumor-infiltrating lymphocytes or chimeric antigen receptor T (CAR-T) cell therapy, which has shown significant outcomes in treating hematological malignancies (Rosenberg et al., 2011; Fry et al., 2018; Depil et al., 2020). Cancer vaccines are one type of immunotherapeutic strategies that have shown promising results in a personalized manner. GVAX is one of the first tested vaccines against pancreatic cancer, and it is composed of the irradiated tumor cell-expressing granulocyte-macrophage colony-stimulating factor (GM-CSF) (Le et al., 2015; Yarchoan et al., 2020).

**FIGURE 1:**
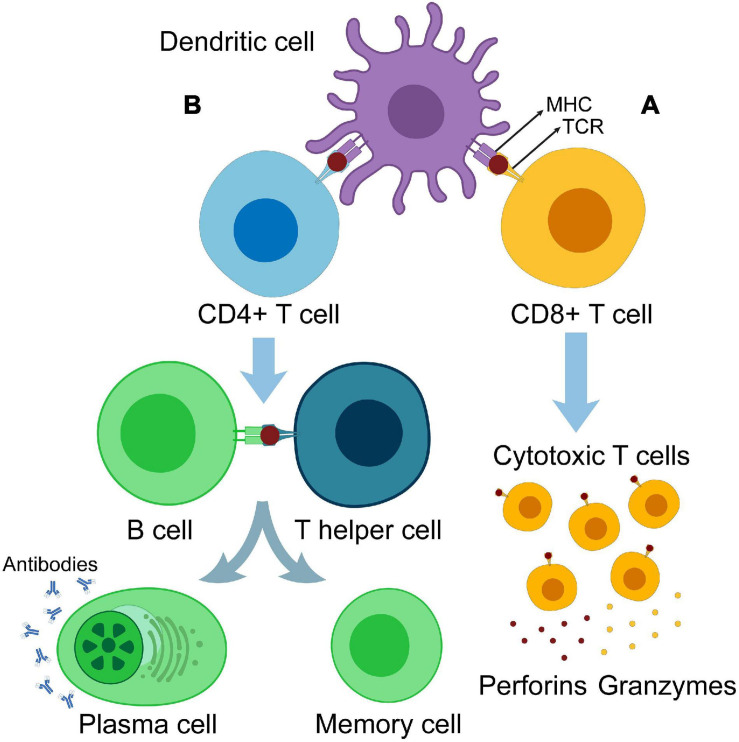
Antigen cross-presentation and T-cell priming. **(A)** Dendritic cell primes CD8^+^ T cells. Primed CD8^+^ cells are differentiated into cytotoxic T cells producing perforins and granzymes. **(B)** DC primes CD4^+^ T cells. Primed CD4^+^ cells are differentiated into T helper cells, which in turn activate B cells and differentiate them into memory cells and antibody-producing plasma cells.

Dendritic cells are a type of innate immune cells and are potent APCs. They play a central role in immune-mediated cancer elimination through antigen presentation and T-cell priming (Steinman, 2007, 2012; Eisenbarth, 2019; Sa-Nunes and Oliveira, 2021). After tumor-associated antigen (TAA) phagocytosis, antigens are processed by two pathways, the cytosolic pathway and vacuolar pathway, by which they are digested into peptides and loaded onto the major histocompatibility complex (MHC) class I. The TAA–MHC I complex is then transported to the DC surface. When DC-presenting TAAs migrate and reach the lymph nodes, they are capable of priming T cells and triggering antitumor immunity (Joffre et al., 2012; Perez and De Palma, 2019). Before maturation, DCs have a high phagocytic capacity. On the other hand, mature DCs have a lower endocytic ability; express greater levels of co-stimulatory molecules [such as cluster of differentiation 80 (CD80), inducible T-cell co-stimulator ligand (ICOSL), programmed cell death ligand-1 (PD-L1), PD-L2, CD31, CD27, and CD70], and C–C chemokine receptor type 7 (CCR7); and secrete high levels of pro-inflammatory cytokines [such as interleukin-12 (IL-12) and tumor necrosis factor-alpha (TNF-α)] (Patente et al., 2018). Accordingly, scientists have developed modified DCs as an effective cancer vaccine approach, leveraging the DCs’ ability to induce both cellular and humoral immunity (Figure 1).

Dendritic cell vaccines were earlier used against highly immunogenic cancers such as melanoma (Nestle et al., 1998; Butterfield et al., 2003). Later on, they have been extensively used in several clinical trials (Table 1). Commonly, patient-derived monocytes or hematopoietic stem cells are collected and differentiated *in vitro* into DCs. Then, DCs are pulsed with TAAs or tumor lysates and cultured with maturation cytokines (Santos and Butterfield, 2018; Yang et al., 2019). This method is hindered by the low amount of immune cells in cancer patients (Tjomsland et al., 2010), which could be a side effect of other treatments such as radiation or chemotherapy. The use of monocyte-derived DCs (MoDCs) obtained from healthy donors can overcome this problem. Other TAA loading strategies include targeting DCs with viral vectors (Goyvaerts et al., 2014; Sharma et al., 2018) and mRNA-engineered DCs (Benteyn et al., 2015; Willemen et al., 2020).

**TABLE 1:** Clinical trials utilizing DC vaccines in cancer immunotherapy.

Intervention	Cancer type	Clinical response	References
Autologous DCs loaded with vaccinia-CEA-MUC-1-TRICOM (PANVAC-V) + autologous DCs loaded with fowl pox-CEA-MUC-1-TRICOM (PANVAC-F)	Resected hepatic or pulmonary metastases of colorectal carcinoma	13 of 16 patients achieved 2 years of recurrence-free survival	Morse et al., 2013
Sipuleucel-T	Prostate cancer	Median OS in treated patients is 25.8 months compared to 21.7 in placebo	Small et al., 2014
Autologous DCs loaded with TAAs	Melanoma	Out of 14 patients, 4 achieved PFS (12–35 months), 5 showed OS (22–40 months), and 4 achieved SD	Schreibelt et al., 2016
TriMixDC-MEL	Melanoma	-71% of the treated patients were alive and disease free vs. 35% of the control -The median time to non-salvageable disease recurrence in treated patients were higher than in control	Jansen et al., 2020
TriMixDC-MEL	Melanoma	-Out of 15 patients, 2 achieved CR, 4 achieved SD, and 7 showed PD -Five out of 15 patients achieved PFS (23.6–34 months)	Wilgenhof et al., 2013
WT1 mRNA-electroporated DCs	Acute myeloid leukemia	-Six of 30 patients achieved CR 107.6 (months median duration), and 19 had a disease relapse -15 of these 19 had a salvage therapy, and 73.3% of them achieved a second CR	Anguille et al., 2017
Autologous DCs loaded with allogeneic non-small-cell lung cancer cells	Non-small-cell lung cancer	-20 of 32 patients were alive 5 years post vaccination -22 of 32 showed immunologic response within 6 months of vaccination	NCT00103116
Peptide-pulsed DCs + indinavir	Ewing’s sarcoma and rhabdomyosarcoma	43% of the treated patients achieved a 5-year OS, and 31% achieved a 5-year EFS	NCT00001566
Adenovirus-p53-transduced DCs + 1-methyl-d-tryptophan	Breast cancer	1 of 21 patients achieved complete response, 7 showed partial response, and 2 achieved stable disease	NCT01042535
CEA mRNA-pulsed DCs	CEA-expressing cancer	3 of 23 showed SD, 1 showed CR, and 18 showed PD	Morse et al., 2003
Tumor mRNA-pulsed DCs	Brain cancer	2 of 5 patients achieved SD, none showed PR, and 3 showed PD	Caruso et al., 2004
Peptide-loaded DCs + dasatinib administered at the same time	Metastatic melanoma	-Four of six patients had partial response, and 2 out of 6 had progressed disease -The calculated ORR in 6 participants is 0.6667	NCT01876212

*OS, overall survival; CR, complete remission; SD, stable disease; PD, progressed disease; PR, partial progression; PFS, progression-free survival; EFS, event-free survival; ORR, overall response rate; CEA, carcinoembryonic antigen.*

Fortunately, after Takahashi and Yamanaka (2006) generated induced pluripotent stem cells (iPSCs) from mouse fibroblasts by introducing four transcription factors (*OCT4*, *SOX2*, *KLF4*, and *c-MYC*), iPSCs have become a template to generate DCs in quantities suitable to produce anticancer efficacy (Senju et al., 2011b; Sachamitr et al., 2014; Kitadani et al., 2018). Furthermore, *in vivo* targeting of DCs, in which nanoparticles, antibodies, viral vectors, and RNA are used as carriers to deliver TAAs, co-stimulatory molecules, or adjuvants to stimulate endogenous DCs (VandenDriessche et al., 2002; Dullaers et al., 2006; Diken et al., 2011; Cubillos-Ruiz et al., 2012; Kreutz et al., 2013; Van Lint et al., 2016), is considered an efficient state-of-the-art approach to bypass leukapheresis inconveniency and the laborious differentiation and maturation protocols. Increasing efforts to understand DC biology will help to derive better DC vaccines either as a single therapy or in combination with other treatment regimens. This review provides a brief overview of the main DC subsets and illustrates how DC and cancer cell crosstalk in the tumor microenvironment (TME) correlates with a positive or negative prognosis. Lastly, we discuss the cutting-edge approaches to using DCs in cancer immunotherapy.

## DC Subsets

DC subpopulations are classified according to their ontogeny, morphology, function, marker expression, and cytokine secretion into three main subtypes: MoDCs, plasmacytoid DCs (pDCs), and conventional DCs (cDCs), which are further divided into type 1 (cDC1s) and type 2 (cDC2s).

### MoDCs

In response to inflammation, monocytes are differentiated into DCs (Collin and Bigley, 2018). MoDCs are generated *ex vivo* in vast amounts from CD14^+^ monocytes or CD34^+^ hematopoietic stem cells through culturing with IL-4 and GM-CSF (Mastelic-Gavillet et al., 2019), which allows for a better understanding of DC biology. T-cell-directed differentiation by DCs is largely dependent on the maturation signal. MoDCs treated with toll-like receptor (TLR) agonists promote Th1 activation. For example, MoDCs electroporated with a polyinosinic: polycytidylic acid [poly(I:C)] analog [poly(I:C12U)], which is a TLR3 agonist, promoted CD4^+^ T-cell expansion and induced their differentiation toward Th1 cells (Kaisho and Akira, 2003; Michiels et al., 2006). Electroporation of MoDCs with CD40L and/or constitutively active TLR4 (caTLR4) encoding mRNA, but not with CD70 mRNA, induces CD4^+^ T-cell differentiation into Th1 cells. However, electroporation of DCs with CD40L, CD70, and caTLR4 mRNA (TriMixDC) in addition to melan A antigen mRNA induces antigen-specific CD8^+^ cytotoxic T cells. Additionally, MoDCs treated with curdlan, a dectin-1 agonist, induce CD4^+^ T-cell skewing toward Th1 and Th17 cells (Bonehill et al., 2008, 2009; Dragicevic et al., 2012). However, *ex vivo* generated MoDCs are transcriptionally distinct from their primary counterparts (Helft et al., 2015), and their migration capacity and efficacy are debated (Morse et al., 1999; Shinde et al., 2018); nevertheless, they remain the cornerstone of cancer vaccine studies due to their accessibility, rapid differentiation, and maturation protocols compared with other subsets (Shinde et al., 2018; Tanyi et al., 2018). Ontogeny studies revealed that inflammatory DCs are the closest phenotype to MoDCs (Segura et al., 2013; Reynolds and Haniffa, 2015). Inflammatory DCs express FcεRI, CD11c, CD11b, CD14, CD1a, and CD209, and they are described in patients with cancer, psoriasis, and atopic dermatitis, and in the synovial fluid of patients with rheumatoid arthritis (Wollenberg et al., 1996; Segura and Amigorena, 2013; Segura et al., 2013).

### pDCs

Plasmacytoid DCs are one type of bone marrow-derived DCs (BMDCs), which arise from common DC precursors and lymphoid precursors (Naik et al., 2007; Geissmann et al., 2010; Rodrigues et al., 2018). pDCs are known for their ability to produce high levels of type I interferon (IFN) upon stimulation of TLR7 and TLR9, and they play a crucial role during viral infections (Reizis et al., 2011; Mitchell et al., 2018). They are characterized by the expression of CD4, CD123, CD303, CD304, blood-derived cell antigen-2 (BDCA-2), human leukocyte antigen-DR (HLA-DR), and TLR7/TLR9 (Swiecki and Colonna, 2015; Villani et al., 2017; Wculek et al., 2020). Matsui et al. (2009) showed that pDCs are further divided into two subtypes based on the expression of CD2, in which the CD2 (high) pDC subpopulation expresses higher levels of CD80 and IL-12 p40. pDCs have limited antigen-presenting potential (Chiang et al., 2016), and their presence in the TME is associated with poor cancer prognosis as they induce tumor progression through stimulation of ICOSL, which in turn stimulates regulatory T (Treg) cells (Conrad et al., 2012; Lombardi et al., 2015). On the other hand, stimulated pDCs have shown promising results as cancer vaccines. In clinical and preclinical melanoma models, different strategies of antigen delivery or loading onto pDCs resulted in significant type I IFN production, antigen-specific T-cell priming, and superior chemoattractive properties to cDC2s, eliciting antitumor activity (Tel et al., 2013; Kranz et al., 2016; van Beek et al., 2020). Conversely, Salio et al. (2004) showed that T-cell priming is independent of type I IFN production. Another study suggested that the presence of pre-classical DCs (pre-CDCs), the intermediate precursors to cDC1s and cDC2s, in the pDC subpopulation might reflect the responsibility for Th1-cell induction and cross-presentation capability (Patente et al., 2018).

### cDC1s

Similar to pDCs, cDC1s, and cDC2s arise from common dendritic progenitors (CDPs) (Patente et al., 2018). cDC1s express CD141, XCR1, and CLEC9A (Poulin et al., 2010; Wculek et al., 2020). They have superior antigen presentation activity on MHC I to cytotoxic T cells (Palucka et al., 2010), thus activating Th1 and CD8^+^ cells (Martinez-Lopez et al., 2015; Laoui et al., 2016). cDC1s have profound antitumor functions, and their presence in the TME correlates with better prognosis and survival rate (Sluijter et al., 2015; Bottcher et al., 2018; Cancel et al., 2019; Zilionis et al., 2019). In this light, the need to generate cDC1s *in vitro* that resemble primary cDC1s in a suitable quantity has gained researchers’ interest. The Notch signaling pathway was identified as a potent inducer of cDC differentiation (Martin-Gayo et al., 2017). Culturing of bone marrow progenitors in a medium containing FMS-like tyrosine kinase 3 ligand (FLT3L) for 3 days followed by co-culturing on monolayers of OP9 stromal cells expressing the Notch ligand Delta-like 1 (OP9-DL1) induced cDC1 differentiation with marker expressions (CD103^+^, Dec205^+^, and CD8α^+^) resembling wild-type cDC1s. The presence of OP9-DL1 produced cDC1s with preferential migration potential compared to other methods (Lee et al., 2015; Kirkling et al., 2018).

*Ex vivo* loading of primary cDC1s with tumor cell lysates induced CD8^+^ and CD4^+^ T-cell infiltration and reduced tumor progression in engrafted tumor models (Wculek et al., 2019). IFN regulatory factor 8 (*Irf8*) (Guilliams et al., 2016) and basic leucine zipper transcriptional factor ATF-like 3 (*Batf3*) (Poulin et al., 2012; Grajales-Reyes et al., 2015) are critical transcriptional factors in the development of cDC1s and are essential for tumor rejection (Theisen et al., 2019). In *Batf3*^–/–^ mice, DCs were not able to mediate rejection of highly immunogenic tumors as they lack cross-presentation potential with subsequent impairment of cytotoxic T-cell activity (Hildner et al., 2008). Transgenic expression of *Irf8* into *Batf3*-deficient mice allowed the development of cDC1s and restored their cross-presentation function. However, these DCs failed to mediate rejection of fibrosarcoma (Theisen et al., 2019). These results indicate that immunogenic rejection of tumors is *Batf3* dependent but not limited to DC ability to cross-present tumor antigens, and there might be other mechanisms involved, such as the ability of cDC1s to communicate with other immune cells through the secretion of CXC-chemokine ligand 9 (CXCL9) and CXCL10, which induce recruitment and infiltration of T cells at the tumor site (Perez and De Palma, 2019). Immune rejection of tumors also lay under the effect of CCR7 expression on cDC1s. CCR7 expression promotes TAA-carrying cDC1 migration to draining the lymph node where CD8^+^ priming occurs, boosting antitumor response (Roberts et al., 2016; Wang et al., 2016).

### cDC2s

cDC2 distribution is found to be lower than that of other DC types. They are characterized by CD172a, CD11c, CD11b, and CD1c (O’Keeffe et al., 2015; Wculek et al., 2020). Like cDC1s, cDC2s have shown antitumor efficacy. They act *via* antigen presentation on MHC II to CD4^+^ T cells, promoting T-cell differentiation into Th1, Th2, and Th17 cells (Leal Rojas et al., 2017; Eisenbarth, 2019). Studies have shown that *Irf4* is essential for cDC2 activity and Th2-cell differentiation, which stimulates humoral immunity and promotes B-cell proliferation (Schlitzer et al., 2013). In mouse models, loss of *Irf4* reduced cDC2 function and defected Th2-cell differentiation (Schlitzer et al., 2013; Williams et al., 2013; Binnewies et al., 2019). In the context of the cDC2 ability to induce antitumor responses, cDC2s were found to efficiently prime CD4^+^ T cells in vaccinated mice and induce Th17-cell differentiation, and most noteworthy, they were able to repolarize tumor-associated macrophages (TAMs) from M2 pro-tumoral phenotype into M1 antitumor phenotype (Laoui et al., 2016). Additionally, cDC2 vaccines pulsed with tumor antigens were tested in clinical trials, and they showed effective and safe antitumor responses against metastatic melanoma and metastatic prostate cancer (Prue et al., 2015; Schreibelt et al., 2016).

## DC Malfunction in Cancer Patients

It is well known that tumors and TME manipulate the immune system to favor their persistence and progression. In the case of colorectal cancer, the presence of elevated numbers of tumor-associated DCs correlated with poor prognosis (Jochems and Schlom, 2011). Several mechanisms were found to perturb DC functions. For example, PD-L1 is highly expressed in tumor-infiltrating DCs, inhibiting T-cell activation and cytokine production. DC activity was restored upon PD-1/PD-L1 blockade (Salmon et al., 2016). Another mechanism is through upregulation of T-cell immunoglobulin and mucin domain-containing-3 (TIM-3) protein on DCs, which inhibits sensing of danger signals (Maurya et al., 2014; de Mingo Pulido et al., 2018). Michielsen et al. (2011) reported that VEGF, CCL1, CCL2, and CXCL5 presence in conditioned medium from colorectal cancer explants inhibited DC maturation and IL-12p production while increasing IL-10 secretion. Melanomas expressing β-catenin were found to induce resistance to immunotherapeutics, reduce infiltrating cDC1s and T cells, and promote tumor growth (Spranger et al., 2015). Moreover, the presence of prostaglandin E2 (PGE2) stimulated tumor growth by impairing the accumulation of intratumoral CD103^+^ DCs (Zelenay et al., 2015).

Metabolic dysfunction can also influence DC maturation in cancer patients. Hypoxia, lactic acid production, and decreased pH impair normal DC function. *In vitro* cultures of prostate cancer or melanoma cells produced high levels of lactic acid, which was associated with modulation of DC differentiation and maturation (Gottfried et al., 2006). Other TME-derived products induce lipid peroxidation, which activates the endoplasmic reticulum stress response factor *via* spliced X-box-binding protein 1, leading to lipid accumulation in DCs (Tyurin et al., 2011; Cubillos-Ruiz et al., 2015). Accumulation of lipid particles inhibits the peptide–MHC I complex migration to the DC surface and impairs cross-presentation potential to T cells, blocking their activity (Herber et al., 2010; Ramakrishnan et al., 2014; Veglia et al., 2017).

Infiltrating pDCs are incapable of type I IFN production and can also stimulate Treg-cell expansion through the expression of indoleamine 2,3-dioxygenase (IDO) and ICOSL, which enhance tumor progression (Ito et al., 2007; Aspord et al., 2013). In many cancer patients, high levels of infiltrating pDCs are linked to poor prognosis (Lombardi et al., 2015; Saadeh et al., 2016). Tumor DCs have shown lower antigen-trafficking potential due to controlled CCR7 expression (Roberts et al., 2016), resulting in decreased ability to prime T cells in lymph nodes. Moreover, signal transducer and activator of transcription 3 (STAT3) phosphorylation, activated by IL-6 and IL-10 in chronic lymphocytic leukemia patients’ sera, induces suppressor of cytokine signaling 5 expression, which in turn inhibits STAT6 activation (an essential molecule for MoDC differentiation), preventing monocyte differentiation and maturation (Toniolo et al., 2016; Kitamura et al., 2017).

## DCs in Cancer Immunotherapy

**FIGURE 2:**
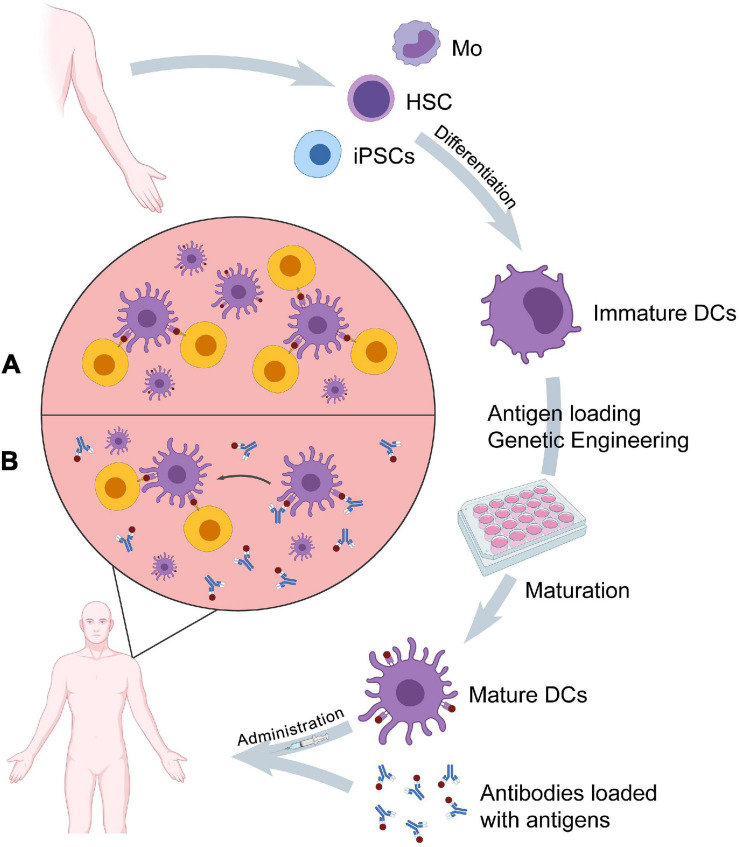
A schematic diagram illustrates the production and mechanism of action of DCs. **(A)** *Ex vivo* generated DCs cross-present tumor antigens to T cells. **(B)** Antibodies transfer loaded TAAs to targeted DCs *in vivo*. MHC: major histocompatibility complex, TCR: T-cell receptor.

As previously mentioned, DCs are the most potent APCs that promote cellular and humoral antitumor immunity, making immunotherapy-based DC vaccines, with either *ex vivo* generated DCs or *in vivo* targeting modalities, an active area of research (Figure 2). That’s why researchers are working to augment their efficacy, providing new paradigms of cancer vaccines, which could be considered as potential candidates in various clinical settings.

### MoDC Vaccines

Based on the understanding of DC biology and their antigen presentation and T-cell activation potential, numerous preclinical and clinical studies utilizing DCs in cancer immunotherapy have been undertaken (Steinman and Banchereau, 2007). Most clinical trials relied on DCs generated *ex vivo* from blood monocytes. Usually, IL-4 and GM-CSF are used to induce DC differentiation from monocytes in 5–7 days (Dauer et al., 2003; Mohty et al., 2003) or 2 days as in the case of FastDCs (Dauer et al., 2005). Other differentiation protocols include culturing peripheral blood mononuclear cells (PBMCs) with IFN-β and either IL-3 or GM-CSF (Breckpot et al., 2005; Mazouz et al., 2005). However, the best maturation cocktail is yet to be defined. Immature DCs have less potential to induce effector immune cells as they do not produce stimulatory cytokines and express less levels of co-stimulatory molecules (Steinman and Swanson, 1995; Trombetta and Mellman, 2005). Different maturation cocktails were tested, such as TLR agonists, CD40 ligand (CD40L), and other cytokines to identify the ideal combination. Vopenkova et al. (2012) have compared various maturation signals’ effect on MoDC functions and stated that lipopolysaccharide and IFN-γ could give the highest response.

To date, the first and only FDA-approved DC vaccine (Provenge) consists of autologous APCs loaded with a recombinant fusion protein antigen, which is composed of GM-CSF and prostatic acid phosphatase (PAP). Provenge synthesis requires 4 days for maturation. It increased the median survival by 4 months in patients with metastatic castration-resistant prostate cancer (Anassi and Ndefo, 2011; Cheever and Higano, 2011). Accordingly, researchers have been developing strategies to acquire DCs with the ability to express TAAs through different techniques (Saxena and Bhardwaj, 2018; Perez and De Palma, 2019). One TAA-loading method is through pulsing DCs with certain epitopes to promote T-cell activity, which was tested in melanoma patients (Carreno et al., 2015). MUC1-pulsed DCs derived from PBMCs were tested in phase I/II clinical trials in patients with resected biliary and pancreatic cancers. MUC1-pulsed DCs increased the survival of 33% of the vaccinated patients for up to 5 years (Lepisto et al., 2008). WT1-pulsed DCs in phase I clinical trials increased antigen-specific cytotoxic T cells in 20% of the treated patients with pancreatic cancer (Yanagisawa et al., 2018). Other TAA-loading strategies include whole-tumor-cell lysate-pulsed DCs (Li et al., 2010; Bauer et al., 2011), which express a broader range of tumor antigens suitable for personalized treatments, tumor cell fused with DC vaccines (Koido et al., 2013; Chen et al., 2015), or genetically modified DCs to express cancer-specific antigens (Miyazawa et al., 2011; Chen et al., 2013; Shindo et al., 2014; Maeda et al., 2015; Esprit et al., 2020).

TriMixDC is one type of mRNA-engineered DCs, which gained researchers’ interest owing to their enhanced antitumor activity and feasibility compared to other mRNA-based vaccines (Anguille et al., 2014; Van Lint et al., 2015). The delivery of CD40L, CD70, and caTLR4 mRNA generates mature DCs in a one-step process without further incubation with other maturation cocktails for a certain period of time, which can exhaust the cells. Moreover, it eliminates the need to perform tumor biopsies followed by further purification in GMP settings for clinical use, as in the case of whole-tumor mRNA-based DC vaccines, which is laborious and time-consuming. Synthetic mRNA-based vaccines produce fewer side effects and show a higher possibility for optimization and large-scale generation than whole-tumor mRNA-based DC vaccines (Van Nuffel et al., 2010; Van Lint et al., 2014; Benteyn et al., 2015). Co-electroporation of TriMixDC with mRNA encoding a fusion of melanoma antigen and DC-LAMP, an approach named TriMixDC-MEL, stimulated antigen-specific CD8^+^ T and Th1 cells in vaccinated patients (Bonehill et al., 2009). In a phase II trial, TriMixDC-MEL combined with ipilimumab, a CTLA-4 inhibitor, showed a 20% complete response and ≃18% partial response in 39 patients with advanced melanoma (Wilgenhof et al., 2016). TriMixDC-MEL plus ipilimumab also resulted in 28% overall survival (OS) after 390 weeks of median follow-up and 18% progression-free survival (PFS) after 5+ years in patients with stage III or IV melanoma (De Keersmaecker et al., 2020).

Additionally, Rosa et al. (2018) reprogrammed mouse and human fibroblasts into DCs named induced DCs (iDCs) by transduction of *PU.1*, *Irf8*, and *Batf3* transcription factors. iDCs have cDC1-like features and can prime antigen-specific CD8^+^ T cells. Furthermore, BMDCs virally transduced with CCR7 gene generated CCR7-overexpressing mature-like DCs, which had a notable migration potential to draining lymph nodes (Okada et al., 2005).

Lastly, Squadrito et al. (2018) engineered DCs expressing chimeric receptors that are able to take up and process TAAs *in situ*. The introduction of the chimeric receptor allowed DCs to selectively take up tumor-derived extracellular vesicles, which can deliver TAAs to DCs. HER2-specific extracellular vesicle-internalizing receptor (EVIR)-expressing DCs showed a significant increase of antigen-specific cytotoxic T cells, resulting in an antitumor response (Squadrito et al., 2018). Blocking immunosuppressive signals is another promising approach in cancer vaccines. Researchers utilized small-interfering RNAs (siRNAs) to knock down PD-L1 and PD-L2 genes in DCs. PD-L-silenced DCs increased T-cell expansion and IFN-γ and IL-12 production (Hobo et al., 2010; van der Waart et al., 2015).

### iPSC-Derived DC Vaccines

Genetically engineered DC vaccines expressing TAAs showed significant effectiveness against many cancer types (Ojima et al., 2007, 2008; Miyazawa et al., 2011). These strategies rely mostly on either primary DCs or *ex vivo* generated MoDCs, which require leukapheresis. Therefore, they are patient inconvenient, and their clinical application is restrained. Also, DCs exist as small populations in the blood (Jongbloed et al., 2010), and their number is further reduced in cancer patients (Beckebaum et al., 2004; Satthaporn et al., 2004; Poschke et al., 2012). Therefore, iPSCs are considered an unlimited and potential source to provide DCs (iPSC-DCs) in a suitable quantity.

Scientists have designed protocols to differentiate mouse and human iPSCs into DCs (Senju et al., 2009, 2011a; Li et al., 2014). For example, Silk et al. (2012) generated CD141^+^XCR1^+^ DCs from iPSCs using a protocol that is free from animal-derived products, making them compatible with clinical applications. These iPSC-DCs were able to cross-present melan A antigen (melanoma antigen) and prime CD8^+^ T cells (Silk et al., 2012). To increase the yield of iPSC-DCs, researchers generated proliferating iPSC-derived myeloid cells (iPSC-pMLs) through the insertion of the *c-MYC* gene into iPSC-derived myeloid cells (iPSC-MLs). iPSC-pMLs were then differentiated into iPSC-DCs through culturing in a medium containing IL-4 and GM-CSF for 3 days. iPSC-pMLs loaded with the OVA257-264 peptide were able to prime CD8^+^ T cells in a syngeneic mouse model. Primed antigen-specific CD8^+^ T cells isolated from mouse spleen killed MO4 cells (OVA-expressing melanoma cells) *in vitro*. OVA257-264 peptide-loaded iPSC-DCs provided immunization for 3 months with no adverse effects (Zhang et al., 2015). To further increase iPSC-pML potency, iPSC-pMLs were virally transduced with the IFN-α gene. In a bilateral melanoma transplantation model, local administration of IFN-α-expressing iPSC-pMLs inhibited the tumor growth at treatment and remote sites, in addition to inhibition of lung metastasis (Tsuchiya et al., 2019).

In another study, researchers produced iPSC-DCs expressing carcinoembryonic antigen (CEA) (iPSDCs-CEA) and stimulated them using a maturation cocktail composed of recombinant human IL-6, IL-1β, TNF-α, and PGE2 for 2 days. iPSDCs-CEA was structurally similar to MoDCs, and the expression levels of CD80 and CD83 co-stimulatory molecules were comparable to those of MoDCs. Mature iPSDCs-CEA and MoDCs produced high levels of IFN-γ and IL-12 with no significant difference in secretion levels between both cell types. Moreover, when iPSDCs-CEA were cultured with different cell lines expressing the HLA-A24 allele (MKN1, MKN45, HT29, and LCL-CEA652 cells), they were able to induce CD8^+^ T cells against MKN45, HT29, and LCL-CEA cells (CEA-expressing cells) but not MKN1 (lacking endogenous CEA). These results indicate that iPSDCs-CEA is able to stimulate human cytotoxic T cells with great specificity against gastrointestinal cancers expressing CEA (Kitadani et al., 2018).

Iwamoto et al. (2014) generated iPSC-DCs and BMDCs. Mature iPSC-DCs expressed high levels of CD80, CD86, CD11c, and MHC II, similar to mature BMDCs. The migratory capacity of mature iPSC-DCs identified by the expression of CCR7 was analyzed and showed comparable results to those of mature BMDCs. In this study, using a gene-based vaccination strategy, researchers imparted both iPSC-DCs and BMDCs the ability to express hgp100 (human melanoma antigen) through transduction with a recombinant adenoviral vector. Tetramer and ^51^Cr-release assays revealed induction of cytotoxic T cells against B16 cells (melanoma cell line) in mice immunized with iPSC-DCs-hgp100 and BMDCs-hgp100. Additionally, iPSC-DCs-hgp100 administration significantly inhibited tumor growth in mice with subcutaneous B16 cells compared to phosphate-buffered saline, iPSC-DCs-LacZ, and BMDCs-LacZ as negative controls, suggesting that iPSC-DCs could be a promising approach in clinical practice as cancer vaccines (Iwamoto et al., 2014).

To enhance the antitumor potential of iPSC-DCs, Mashima et al. (2020) generated proliferating and GM-CSF-producing myeloid cells (GM-iPSC-pMLs) through the insertion of *Csf2* and *c-MYC* genes into iPSC-MLs by a lentivirus vector. Similar to BMDCs, GM-iPSC-pMLs were able to stimulate cytotoxic T-cell proliferation. Additionally, when GM-iPSC-pMLs were pulsed with an OVA peptide, they were able to prime and stimulate antigen-specific cytotoxic T cells, indicating that GM-iPSC-MLs had cross-presentation capacity like DCs. Interestingly, in a prophylactic experiment, administration of GM-iPSC-MLs loaded with the OVA peptide were able to inhibit tumor growth when taken 7 days before the mice were injected with subcutaneous MO4 cells.

### Stimulation of DCs *in vivo*

Since *ex vivo* generated MoDCs have limited migration potential, it is crucial to focus on other research lines that involve systemic activation of *in vivo* DCs. Historically, researchers used immune activators such as bacterial products (Coley, 1910; Bernardes et al., 2010), TLR agonists (Adams, 2009; Chi et al., 2017), and bacillus Calmette–Guérin (BCG) (Kamat et al., 2017) to elicit antitumor activity. Immune activators have been found to induce antitumor immune response *via* DC activation (Kuhn et al., 2013) followed by CD8^+^ T-cell priming (Kuhn et al., 2015), and this approach is likely only functional when acting on DCs that already acquired tumor antigens, such as tumor-associated DCs. TGF-β (Pu et al., 2018), 1-methyl-tryptophan (IDO inhibitor) (Li et al., 2010), and inhibiting IL-10 antibody (Marvel and Finn, 2014) have proven to act synergistically with DC vaccines in inhibiting pancreatic cancer growth. Intratumoral injection of cyclic diguanylate monophosphate (STING agonist) or cytosine-phosphorothioate-guanine oligodeoxynucleotide (TLR agonist) enhanced T-cell activation and stimulated *in situ* DC maturation (Kawarada et al., 2001; Ohkuri et al., 2014). Imiquimod, a TLR7/TLR8 agonist, promotes pDC-mediated antitumor activity, and it is approved for the treatment of non-melanoma skin cancer (Drobits et al., 2012). Poly[I:C] and its derivatives have been used in different cancer vaccination studies and have shown significant outcomes (Martins et al., 2015).

In a melanoma mouse model, co-administration of Poly[I:C] and FLT3L enhanced CD103^+^ DC expansion and CD8^+^ T-cell recruitment at the tumor site and synergized PD-L1 antitumor activity (Salmon et al., 2016). The FDA granted an orphan drug designation to a rabies vaccine combined with poly[I:C], named YS-ON-001, for the treatment of pancreatic cancer and hepatocellular carcinoma (Goyvaerts and Breckpot, 2018). Importantly, in an ovarian cancer model, *in situ* co-administration of CD40 and TLR3 agonists has induced the polarization of tumor-infiltrating DCs into an immune stimulatory phenotype that was able to produce type I IFN and IL-12 p70, resulting in tumor remission (Scarlett et al., 2009). Interestingly, Penafuerte et al. developed FIST, a fusion protein of IL-2 and the ectodomain of TGF-β receptor II, to block immunosuppression activity of locally secreted TGF-β and to activate IL-2 receptor-expressing lymphocytes. Administration of this fusokine recruited immune cells at the tumor site and stimulated IFN-γ secretion (Penafuerte and Galipeau, 2012). Likewise, Van der Jeught et al. (2014) developed mRNA encoding IFN-β and the ectodomain of TGF-β receptor II fusokine, named Fβ2. When this mRNA was taken up by DCs and translated into the functioning protein, it stimulated DCs and induced antitumor immunity. Other strategies for intratumoral delivery of immunostimulatory signals such as TNF-α, IL-12, and TGF-β and IL-10 neutralization are extensively reviewed elsewhere (Van der Jeught et al., 2015).

Cancer stem cells (CSCs) play a critical role in cancer progression and metastasis. CSCs are resistant to treatment since they possess antigens different from those present in differentiated tumor cells (Reya et al., 2001). Therefore, vaccination strategies relying on cells expressing stem cell antigens have gained researchers’ interest (Dashti et al., 2016; Zhao et al., 2017). For instance, scientists have developed next-generation cancer vaccines that are more potent and targeted than conventional treatments. Mackiewicz et al. (1995) have developed a genetically engineered whole-tumor cell vaccine expressing hyper-IL-6 against melanoma, named AGI-101H, which has a melanoma stem cell-like phenotype (Mackiewicz and Mackiewicz, 2009). In clinical trials, this vaccine increased the survival of patients with advanced-stage melanoma (Mackiewicz et al., 2015, 2018). Genetically modified B16F10 (melanoma cell line) expressing hyper-IL-6 mixed with murine iPSCs increased DCs, natural killer (NK)-cell infiltration, and IFN-γ and IL-12 p70 production at the tumor site in a mouse model. The vaccines also inhibited the number of infiltrating Treg cells at TME and increased serum level of specific IgG against tumor cells, resulting in a significant reduction of tumor growth with a subsequent increase in the survival rate of the treated mice (Gabka-Buszek et al., 2020).

Targeting of DCs *in vivo* is another strategy that has shown promising results. DEC205 and CLEC9A are receptors that only DCs express. Antibodies targeting these receptors are efficient delivery molecules (Kreutz et al., 2013; Tullett et al., 2016). Mahnke et al. (2005) conjugated melanoma antigens with a DEC205 antibody. The conjugate selectively delivered the neoantigens to DCs, which stimulated CD4^+^ and CD8^+^ T-cell responses, leading to tumor regression (Mahnke et al., 2005). In a phase 1 clinical trial, anti-DEC-205 antibody-mediated delivery of NY-ESO-1 antigen was found to be safe and immunogenic, and it was tolerable as a combination therapy with immune checkpoint inhibitors (Dhodapkar et al., 2014). Conjugation of MUC1 antigen to oxidized mannan targeting mannose receptors (MRs) on DCs also stimulated DCs, and it is 1,000 times more efficient than reduced mannan conjugated to MR in MHC I presentation to cytotoxic T cells (Apostolopoulos et al., 2014). Researchers have used nanoparticles as vehicles in cancer immunotherapy to deliver synthetic long peptides (SLPs), mRNA, or viral vectors to overcome the drawbacks of protein-based vaccines, such as their limited cellular uptake and susceptibility to degradation by enzymes (Varypataki et al., 2016; Verbeke et al., 2017, 2019; Sharma et al., 2018).

Poly[I:C]-adjuvanted SLPs covalently bound to cationic dextran nanogels facilitated peptide internalization into DCs and stimulated cytotoxic T-cell response *in vivo* (Kordalivand et al., 2019). Similarly, SLPs loaded into cationic liposomes and adjuvanted with TLR ligand efficiently induced antigen-specific T cells *in vivo* (Varypataki et al., 2016). In the case of RNA-loaded nanoparticles, Kranz et al. (2016) used lipoplexes as carriers to protect RNA encoding neoantigens from ribonuclease degradation and efficiently deliver RNA to DCs. RNA lipoplexes were tested in clinical trials and found to induce IFN-α production and stimulate effector and memory T-cell activity (Kranz et al., 2016). Cubillos-Ruiz et al. (2012) designed a nanoparticle carrying a Dicer substrate, which mimics endogenous pre-miRNA. Uptake of this complex significantly induced miR-155 activity and reverted the tolerogenic potential of tumor-associated DCs. Subsequently, the complex abolished ovarian cancer progression in 33% of the treated mice (Cubillos-Ruiz et al., 2012). Lentiviral vectors attached to nanobodies is another strategy that has proven to have an efficient targeting potential to DCs both *in vivo* and *in vitro* (Goyvaerts et al., 2012). Combination therapy of plasmids carrying complementary DNA for FLT3L and adenoviral vector carrying IL-18 gene induced DC mobilization with higher CD86 expression, and achieved complete eradication of MCA205 fibrosarcoma in tumor-bearing mice (Saito et al., 2008).

## Conclusion

In the past years, immunotherapy has proven to be an off-the-shelf treatment approach in oncology due to its higher specificity and targeting capacity compared to traditional treatments, including, but not limited to, adoptive transfer of (NK) cells (Burger et al., 2019), macrophages (Klichinsky et al., 2020), T cells (June et al., 2018), and DCs.

Dendritic cells are a heterogeneous type of cells and play a vital role in maintaining immune homeostasis. They are known as environmental sensors and are efficacious in phagocytizing non-self-antigens and presenting them on MHC I and II to CD4^+^ and CD8^+^ naïve T cells. As a result of their plasticity, they are greatly affected by tumor-derived products. Thus, combinatorial strategies with other treatment modalities may act synergistically to inhibit DC tolerogenic polarization and improve their anticancer effect.

Dendritic cell vaccines are found to be feasible, safe, and immunogenic in clinical trials, making them an active area of research. For example, TriMixDC-MEL has shown promising results in inducing antitumor immunity, and it is being tested in clinical trials against melanoma. Moreover, strategies to deliver antibody-loaded neoantigens, activation signals, or nanobodies carrying SLPs or mRNA to induce DC activation *in vivo* have proven their efficacy in preclinical and clinical settings. On the other hand, some DC vaccine approaches have shown suboptimal antitumor activity, which could be due to improper DC generation protocol, maturation cocktail, or route of administration. To date, we do not know which DC subset, maturation cocktail, or antigen loading strategy is ideal for producing optimal efficacy. Notably, all DC subsets contribute to antitumor immunity. That’s why a better understanding of DC biology could pave the way to developing multiplexed DC vaccines, leveraging the crosstalk among DC subpopulations.

Interestingly, the advancement in stem cell-based research provided a template for the development of personalized iPSC-DC vaccines. Furthermore, targeting strategies of DCs *in vivo* to selectively deliver molecules to certain primary DC subsets offer a substitute for the laborious, time-consuming, and costly *ex vivo* generation, antigen loading, and maturation of DCs. Overall, taking into consideration the pros and cons of DC vaccines, it remains tempting to continue researching this field, aiming to provide innovative strategies to enhance their clinical efficacy.

## Author Contributions

AS, NQ, and YW contributed to the study conception and design. AS, HW, YL, MJ, and W-BO performed data collection and analysis. AS wrote the first draft of the manuscript. All authors read and approved the final manuscript.

## Conflict of Interest

HW and NQ were employed by the company Hangzhou Biaomo Biosciences Co., Ltd., Hangzhou, China and Asia Stem Cell Therapies Co., Limited, Shanghai, China. MJ was employed by the company Hangzhou Biaomo Biosciences Co., Ltd., Hangzhou, China. The remaining authors declare that the research was conducted in the absence of any commercial or financial relationships that could be construed as a potential conflict of interest.
